# Finding the fragments: community-based epidemic surveillance in Sudan

**DOI:** 10.1186/s41256-023-00300-7

**Published:** 2023-06-09

**Authors:** Mona Ibrahim, Nada Abdelmagid, Rahaf AbuKoura, Alhadi Khogali, Tasnime Osama, Aljaile Ahmed, Israa Zain Alabdeen, Salma A. E. Ahmed, Maysoon Dahab

**Affiliations:** 1 Department of Social Policy and Intervention, University of Oxford, Oxford, UK; 2 Department of Infectious Disease Epidemiology, London School of Hygiene and Tropical Medicine, London, UK; 3 Julius Global Health, University Medical Centre Utrecht, Utrecht, The Netherlands; 4 National Ribat University, Khartoum, Sudan; 5 Department of Primary Care & Public Health, Imperial College London, London, UK; 6 Sudan COVID-19 Research Group, Khartoum, Sudan; 7 University of Khartoum, Khartoum, Sudan; 8 Freelance Public Health Researcher, Sydney, Australia; 9 Department of Infectious Disease Epidemiology, London School of Hygiene & Tropical Medicine, London, UK

**Keywords:** Global health security, Epidemic surveillance, Health policy, Low-income country, Community-led surveillance

## Abstract

Sudan faces inter-sectional health risks posed by escalating violent conflict, natural hazards and epidemics. Epidemics are frequent and overlapping, particularly resurgent seasonal outbreaks of diseases such as malaria, cholera. To improve response, the Sudanese Ministry of Health manages multiple disease surveillance systems, however, these systems are fragmented, under resourced, and disconnected from epidemic response efforts. Inversely, civic and informal community-led systems have often organically led outbreak responses, despite having limited access to data and resources from formal outbreak detection and response systems. Leveraging a communal sense of moral obligation, such informal epidemic responses can play an important role in reaching affected populations. While effective, localised, and organised—they cannot currently access national surveillance data, or formal outbreak prevention and response technical and financial resources. This paper calls for urgent and coordinated recognition and support of community-led outbreak responses, to strengthen, diversify, and scale up epidemic surveillance for both national epidemic preparedness and regional health security.

## Background

High-level commitments towards global health security gained significant momentum across Africa since 2021, fortifying and further expanding on post-Ebola resolutions from 2014. However, many low-income and fragile settings are not able to fulfil these ambitions due to under-resourced and fragmented epidemic surveillance systems and weak integration to outbreak responses. This paper considers the embodiment of these challenges in Sudan, a country experiencing compounding political, economic, and social fragility for decades. Responding to the recent spikes of deadly outbreaks since 2020—including Dengue, Cholera, Rift Valley fever, COVID-19 and Malaria outbreaks—it is vitally important to examine the strengths and downfalls of Sudan’s disease surveillance systems and the role are, or should, play in national and regional health security.

Sudan is the third largest country in Africa and home to over 45 million people. While a significant proportion of people live in urban settings, many are hard-to-reach presenting a challenge to communicable-disease surveillance and outbreak response efforts. Despite significant national efforts to improve epidemic surveillance in Sudan, particularly in crisis-affected areas, rural communities remain underserved. This paper examines the gaps in formal and informal community-level epidemic surveillance systems, and their implications on outbreak responses.

## The epidemic risk: The Sudanese tale of recurring and escalating threats

In recent years, Sudan has seen increasing frequency and severity of infectious disease outbreaks, many classified as pandemic-causing pathogens by the United Nations [1]. Seasonal, and often concurrent, outbreaks are frequent across the country, with worsening severity and expansion to new geographical areas. This includes malaria, cholera, dengue fever, and chikungunya. During 2020 alone, Sudan reported 706 cases of suspected polio, 81 cases and one death due to dengue fever, 599 cases of suspected chikungunya, and an alarming 1.9 million cases of malaria—with the incidence of malaria exceeding epidemic thresholds in all parts of the country [2]. Despite an already high risk of epidemic vulnerability, COVID-associated budget reallocations and escalating armed conflict exacerbated epidemic risks to new heights.

With increasing outbreaks across the country, most with inadequately defined etiologies, Sudan’s national outbreak surveillance system has struggled to keep up with overwhelming needs for outbreak confinement. On review, there is a scarcity of public information on the system’s performance, with the most recent public government-led evaluation dating back to 2007 [3]. The, likely outdated, system remains divorced from epidemic responses and prevention. Considering the frequent and worsening economic shocks in Sudan, it is likely the national epidemic surveillance is effectively crippled by a critically underfunded public service system. Consequently, Sudan is a fertile ground for the relapse, mutation, and metastasization of infectious diseases and pandemic-capable illnesses [1]. This potentiates hidden and protracted spread of infections nationally across Sudanese states and regionally across the poorly-regulated Sudanese borders—as seen in the COVID-19 pandemic [4].

This gap in both evidence and operations is more apparent at the community level. The verticality of surveillance programmes, staff shortages, and resource limitations may prompt surveillance officers to disproportionately focus on disease-specific surveillance. As a result, initial epidemic responses are often fragmented from national responses. Communities have often become the first responders, especially outside urbanised areas. In Sudan, community-led responses have extended beyond outbreaks to relieve other localised disasters like flooding [5]. Understanding both the informal responses to epidemics is critical to achieving national health security goals.

## National epidemic surveillance: piecing the puzzle

**Fig. 1:**
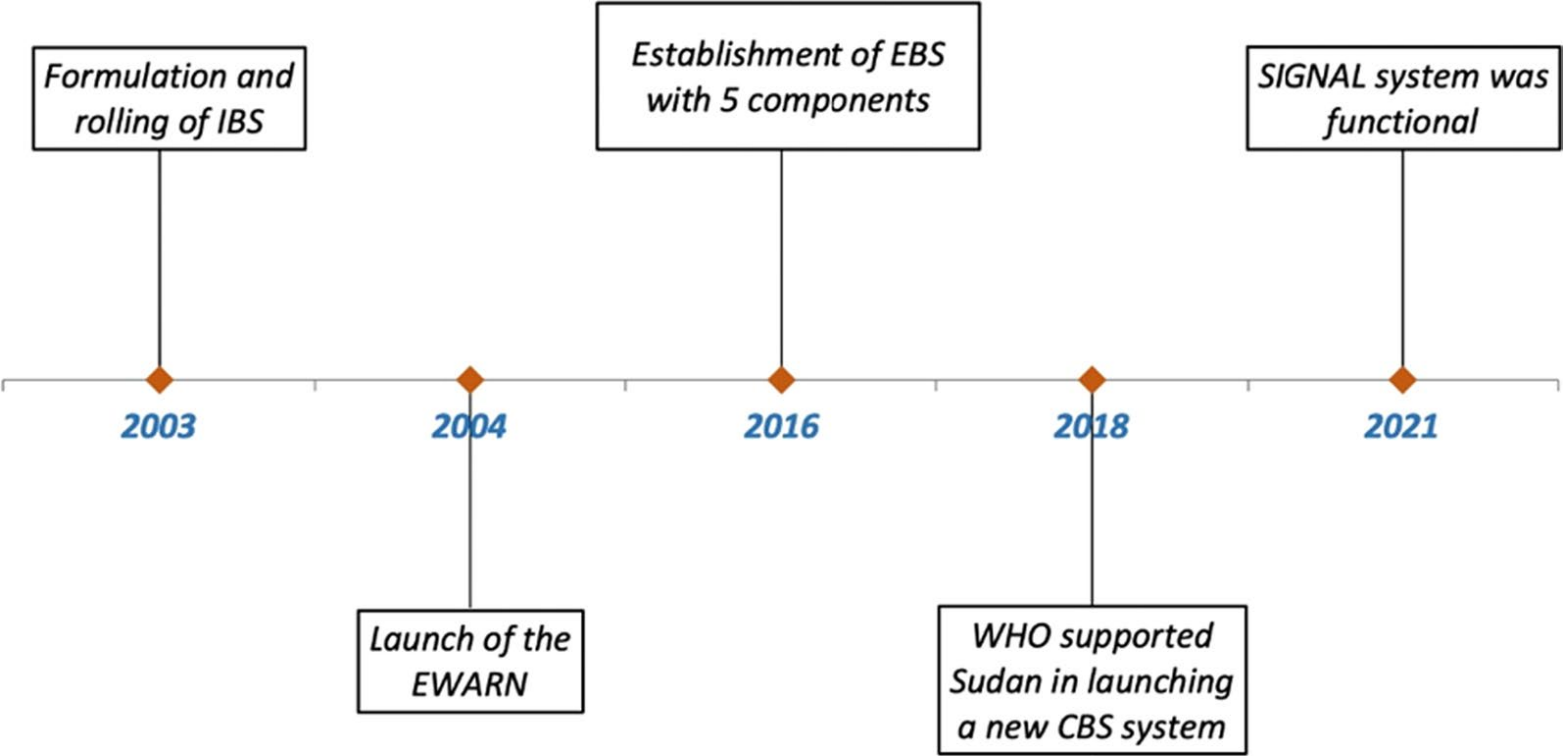
A timeline of recorded events relating to national epidemic surveillance systems in Sudan

The origin of today’s IBS system in Sudan traces back to a re-organisation of the system in 2003 (Fig. 1), which initially included 22 notifiable health events. Despite changes over the years, it remained a passive sentinel surveillance system, covering approximately 30% of health facilities. During outbreaks, the system expands into active surveillance. Evaluations of this system in Khartoum state—a better-resourced state—report long-standing drawbacks relating to timeliness, completeness and accuracy of reporting [6].

Sudan has a history of intertwined conflicts and disease outbreaks [7], with most international funding going into humanitarian responses; naturally, epidemic surveillance systems seem to be linked to humanitarian response efforts. In 2004, early reports of Darfur’s accelerating epidemic risks prompted the launch of an EWARN. EWARN consisted of health-facility based epidemic surveillance, with a rumour verification component for events reported by key informants in the affected communities [6]. It is unclear how well the EWARN system functions today, whether connected to the earlier IBS system, or to the later established Event-based surveillance (EBS).

EBS, established in 2016, consists of five distinct components: (i) Community event-based surveillance (CEBS), (ii) National Hotline and Media Scanning, (iii) Point of Entry, (iv) Partner event-based surveillance by non-health actors, and (v) Health-facility event-based surveillance. However, in reality, CEBS is the only component of EBS that is functioning at the state level [8].

There are numerous challenges in the organization and implementation of these systems. For example, CEBS is notably heavily reliant on volunteer staff for core epidemic information functions; this leaves the system vulnerable to volunteers being demotivated, poorly incentivised, overworked, and underprepared. In 2018, the WHO supported Sudan in launching a new community-based disease surveillance system, which provided a base for the SIGNAL system that followed in 2021, but whether this system complements the existing CEBS system still remains unclear across public documents [8]. Scarcity of publicly available documents makes it difficult to evaluate any of the surveillance systems.

There is also clear fragmentation of the surveillance data amongst the different directorates of the Ministry of Health. For example, surveillance of vaccine-preventable epidemic-prone diseases is routinely managed separately from cholera or malaria surveillance. This is likely associated with limited, and often disease-specific, funding for surveillance. Even when a disease outbreak is detected, the Ministry of Health (MoH) is not guaranteed to act on it due to political and economic disincentives. Reportedly, trade advancement and exports have often taken precedence over epidemic responsiveness [9].

## The informal system: crowdsourcing epidemic surveillance

Sudanese communities are often the first, and possibly main, epidemic responders; they retain a wealth of understanding on local epidemic-prone diseases and how to contain them [10]. Mounting and sustaining this type of independent community-led response can be burdensome on localities. Albeit, their long-standing and locally-acquired knowledge allows for much needed visibility on emerging community outbreaks. These interweaved civic networks can supplement national blind spots to epidemics in Sudan.

Community-led response to localised or national disasters in Sudan is deeply rooted in the concept of ‘Nafeer’, which means ‘call to action’ and holds a unique space in Sudanese culture. In 2013, for example, youth volunteers launched the ‘Nafeer initiative’ to respond to severe flood damages in Khartoum—conducting needs assessments immediately before mobilising resources, responding weeks before an official government response [5]. Although this type of response is well known locally, it is not well documented, preventing distant observers from learning from it. Another notable example is the Sudan Youth Peer Education Network (Y-PEER), consisting of 2000 activists working across 18 of the 19 Sudanese states.

## Recognising the responders: a call for inclusive community-level surveillance systems

Realising informal epidemic surveillance systems is the first step towards resourcing, bridging, and leveraging the civil responses in Sudan. This paper calls for an urgent diversification of outbreak governance, leadership, and coordination structures to include non-governmental actors and meaningful community representation—a necessary shift towards human-centric outbreak surveillance systems. This includes reform across the following dimensions:

1. *Inclusive high-level coordination*. This should start with the necessary bridging of the humanitarian and routine surveillance systems in Sudan. In order to diversify decision-making at both federal and state levels, it is vital that the design and implementation of surveillance systems is inclusive of civil society and first-responders, thus more community-responsive. This starts with streamlining funding steams, through donor and government coordination mechanisms, to implement unified community-level surveillance systems.
2. *Scale-up and popularisation of community-level surveillance systems*. A systematic mapping of existing community-level epidemic surveillance systems is necessary, especially in expanding the recognition and functionality of community information systems set up during the COVID-19 pandemic. The Sudanese MoH has long considered a shift from disease-specific responses to integrated outbreak response plans; it is important that this shift includes both formal and informal community healthcare workers, who can be appropriately compensated, retained, and supported in upholding community-level outbreak reporting.
3. *Bridging surveillance to preparedness*. Operationalisation of outbreak data can inform seasonal epidemic prevention, community resilience building, and targeted health provisions to areas experiencing recurrent, worsening, but predictable, outbreaks. An integrated database can allow for intra- and inter-ministerial responses to mitigate the determinants of disease—including both MoH programmes (e.g. vaccination campaigns), and wider government services (e.g. social protection, sanitation and food security). Further, academic and civic representation in both the design and analysis of surveillance data can strengthen the ministry’s capacity to anticipate outbreaks across all levels, and allows for civic response integration in the future.

## Conclusions

Learning from the downfalls that led to the COVID-19 pandemic, countries like Sudan—suffering high burdens of infectious diseases—must put in place the necessary precautions to prevent epidemics. Investments into collaborative community-level surveillance can contribute to efficient outbreak diagnostics, responses, disaster risk reduction, and long-term preparedness.

## Data Availability

Not applicable.

## Abbreviations

IBS: Indicator-based epidemic surveillance
EWARN: Early warning alert and response system
EBS: Event-based surveillance
CEBS: Community event-based surveillance
MoH: Ministry of health
